# Dual-polarity metamaterial circular polarizer based on giant extrinsic chirality

**DOI:** 10.1038/srep16666

**Published:** 2015-11-12

**Authors:** J. H. Shi, Q. C. Shi, Y. X. Li, G. Y. Nie, C. Y. Guan, T. J. Cui

**Affiliations:** 1 Key Laboratory of In-Fiber Integrated Optics of Ministry of Education, College of Science, Harbin Engineering University, Harbin 150001, China; 2 State Key Laboratory of Millimeter Waves, Southeast University, Nanjing 210096, China

## Abstract

Chirality is ubiquitous in nature. The associated optical activity has received much attention due to important applications in spectroscopy, analytical chemistry, crystallography and optics, however, artificial chiral optical materials are complex and difficult to fabricate, especially in the optical range. Here, we propose an ultrathin dual-polarity metamaterial circular polarizer by exploiting the mechanism of giant extrinsic chirality. The polarity of the circular polarizer with large suppression of linear anisotropy can be switched by changing the sign of incident angle. The microwave experiments and optical simulations demonstrate that the large angle of incidence facilitates the high-efficiency circular polarizer, which can be realized in the whole spectra from microwave to visible frequencies. The ultrathin single-layer metamaterials with extrinsic chirality will be a promising candidate for circular polarization devices.

An object is chiral if it cannot be superposed onto its mirror image. It has different responses to the right circularly polarized (RCP) and left circularly polarized (LCP) waves. The related chiral effect - optical activity - has received much attention due to important applications in spectroscopy, analytical chemistry, crystallography and optics^1^. Optical activity has two manifestations of circular birefringence and circular dichroism. Circular birefringence refers to the ability to rotate the polarization state of light, while circular dichroism is differential transmission of circularly polarized waves. Although chirality is ubiquitous in nature, chiral optical materials are usually bulky along light propagation direction compared with the wavelength due to their weak response. Recent advances of metamaterials provide a promising route to achieve strong artificial chirality and anisotropy for manipulating the interaction between light and chiral meta-molecules^2^,^3^,^4^,^5^,^6^,^7^,^8^. Since the realization of negative refractive index in chiral metamaterials was successfully completed^9^,^10^,^11^, great efforts have been devoted to achieving giant circular dichroism^12^,^13^,^14^, optical activity^15^,^16^,^17^,^18^ and asymmetric transmission^19^,^20^,^21^,^22^. Especially, a series of chiral devices have been achieved for practical applications such as the optical vortex generator^8^, polarization rotator^23^,^24^, linear and circular polarizer^25^,^26^,^27^, polarization spectrum filter^28^,^29^ and chiral switching^30^,^31^.

The newly observed asymmetric transmission arises from direction-dependent circular conversion dichroism in so-called 2D or planar chiral metamaterials and exhibits itself as a difference in total transmission levels for two opposite propagation directions^19^, completely different from optical activity in metamaterials with 3D chirality that are constant for two opposite propagation directions^15^. Initially, optical activity and asymmetric transmission were regarded as inherent characteristics of intrinsically two- and three-dimensional (3D) chiral metamolecules, respectively. The recent endeavors have verified that the aforementioned chiral effects can also be observed in intrinsically non-chiral metamaterials^15^,^32^,^33^,^34^,^35^,^36^,^37^, provided that the whole arrangement formed by such metamaterials together with the wave vector of light cannot be superposed upon its mirror image. This phenomenon was termed as extrinsic chirality, where two requirements are necessary, i.e., metamolecules without 2-fold rotational symmetry and the oblique incidence^15^. When the incidence plane does not contain the mirror line of the metamaterial, the whole system is chiral. Strong chiral effects are indistinguishable from those of chiral three-dimensional media. The strengths of optical activity and asymmetric transmission can be easily tuned by the angle of incidence^33^. Following the previous work by Plum *et al.*^15^, the linear polarization conversion in the extrinsic chiral metasurface was numerically studied to experience a change from a single ripple-free peak to a spectrum splitting with two separated peaks at a grazing incidence^37^. In particular, such a metasurface can be expected as a circular polarizer to distinguish between RCP and LCP waves^15^,^37^. In fact, much attention has been paid to the optical activity in extrinsically chiral metamaterials^15^,^32^,^33^, in which, however, the incident angle was limited to less than 30°. Due to the linear anisotropy, the extrinsically chiral metamaterial exhibits low circular transmittance and relatively large circular-polarization conversion^15^, which hamper the performance of the circular polarizer. The possible scheme to achieve circular polarizer using extrinsically chiral metamaterials has not been studied^15^. Here, we numerically and experimentally demonstrate a simple theme to realize a high-efficiency circular polarizer, where the large angle of incidence leads to a weak linear anisotropy and the circular polarization transmittance is nearly 90%. The underlying circular polarizer is not based on intrinsic chirality, but on extrinsic chirality. More importantly, the left and right polarity of such high-efficiency polarizer can be interchangeable by changing the incident angle. The single-layer metamaterial with extrinsic chirality is easy for fabrication. The dual-band circular polarizer can be engineered to be anywhere from the microwave to visible frequencies and may be integrated with terahertz and nanophotonic systems.

In this article, we experimentally demonstrate a dual-polarity metamaterial circular polarizer by exploiting the mechanism of giant extrinsic chirality. The metamaterial is constructed by an array of asymmetrically split ring apertures (ASRAs) perforated through a metallic film of subwavelength thickness. The polarity of the circular polarizer with largely suppressed linear anisotropy can be interchanged by changing the direction of the grazing incidence. Remarkably, microwave experiments and optical simulations demonstrate that the strong circular dichroism and birefringence can be tailored to be anywhere from microwave to optical frequencies. Such the single-layer metamaterial with extrinsic chirality will be a promising candidate for diverse circular polarization functionalities.

## Results

### Metamaterials design and theory

**Figure 1:**
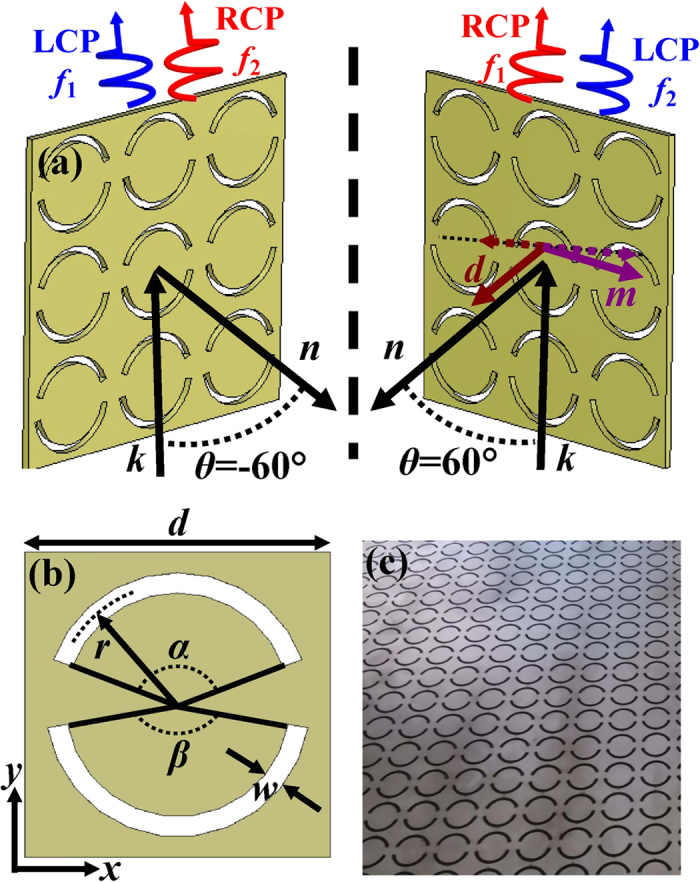
Schematic of an metamaterial circular polarizer. (**a**) Extrinsic chirality. The polarity of the circular polarizer can be interchangeable by changing the sign of the angle of incidence. The dashed arrows indicate projections of the induced electric dipole moment *d* and magnetic dipole *m* onto the plane perpendicular to the wave vector. (**b**) The structure of a unit cell. The metamaterial consists of an array of asymmetrically split ring apertures (ASRAs). Each ASRA resonator has two different metallic apertures corresponding to arc angles *α* and *β*. (**c**) The front view of a metamaterial sample.

The ASRA meta-molecule being complementary to asymmetrically split ring (ASR) is an important building block of metamaterial structures for achieving trapped-mode resonance, optical activity and asymmetric transmission as well as extrinsic chirality^15^,^37^. At oblique incidence, an achiral planar structure and the direction of incidence can form an extrinsically 3D-chiral spatial arrangement shown in Fig. 1(a). The angle of incidence *θ* is measured between the wave vector *k* and the metamaterial’s surface normal *n*. According to Babinet’s principle, the magnetic dipole (*m*) in the plane of the ASRA metamaterial and the electric dipole (*d*) perpendicular to the plane of the ASRA metamaterial are alternatively induced when a wave polarized along the mirror line of the ASRA is incident. As the oblique incidence occurs, there are identically oriented projections of the corresponding dipole moments onto the plane perpendicular to the wave vector indicated by dashed arrows in Fig. 1(a), thus resulting in the cross coupling. The chiral metamaterial exhibits distinctly different responses to the RCP and LCP waves when the extrinsic chirality is strong. For some certain angle of incidence, maximum optical activity occurs when the aperture is parallel to the plane of incidence^15^. It is transparent to the RCP wave at one frequency *f*_1_ and the LCP wave at the other frequency *f*_2_ in the case of *θ* > 0° while transparent to the RCP wave at *f*_2_ and the LCP wave at *f*_1_ in the case of *θ* < 0°, as shown in Fig. 1(a). Chiral effects of opposite signs are readily created by angles of incidence of opposite directions^15^. Therefore, it is anticipated that the ASRA metamaterial can work as a dual-polarity circular polarizer.

In this work, we propose a non-chiral metamaterial composed of an array of square ASRA sketched in Fig. 1. The ASRAs are periodically perforated through a freestanding metal plate with a thickness of 1 mm. The period of perforation is *d* = 15 mm. Each ASRA consists of two different arc slits corresponding to open angles α = 140° and β = 160°, as shown in Fig. 1(b). The radius of the ASRA is *r* = 6 mm and the slit width is *w* = 1 mm. Figure 1(c) shows the picture of the fabricated sample. Numerical simulations were performed by use of the commercial software CST microwave studio. A lossy metal was simulated with its conductivity of 1 × 10^7^ S/m. The transmission properties of the metamaterial were studied for both linearly and circularly polarized waves in the frequency range from 8 to 12 GHz. The calculated results can be presented in terms of transmittance *T*_*ij*_ (*T*_*ij*_ = |*t*_*ij*_|^2^). The subscripts *i* and *j* correspond to the polarization states of the transmitted and incident waves, which could be either x and y linearly polarized wave or + and − circularly polarized wave. The circular transmission matrix can be calculated from the linear one by Eq. (1)


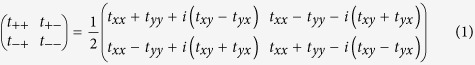


where “+” and “−” denote RCP and LCP waves, respectively. The polarization rotation azimuth angle *φ* and ellipticity *η* are calculated by Eq. (2)


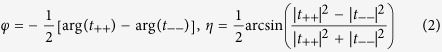


### Simulated and measured results

**Figure 2:**
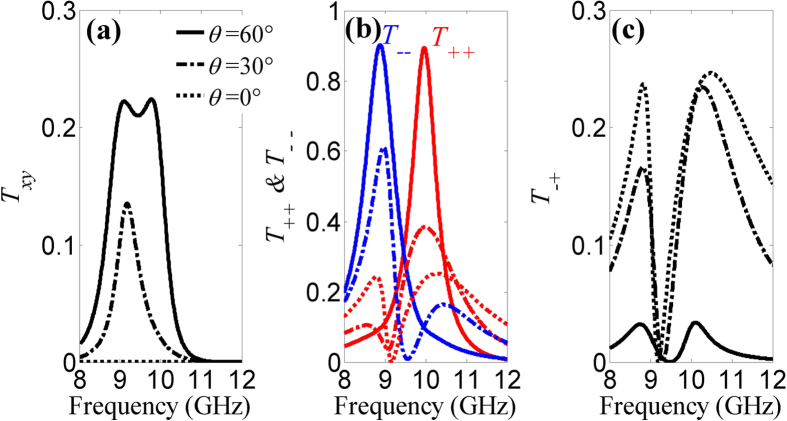
Simulated transmittance spectra of the metamaterial at *θ* = 0°, 30° and 60°. (**a**) Linear polarization conversion, (**b**) circular transmittance and (**c**) circular polarization conversion. Dashed line −0°, dash-dotted line −30° and solid line −60°. *T*_++_ and *T*_−−_ coincide with each other at normal incidence.

Figure 2 shows simulated transmittance spectra of the ASRA metamaterial at *θ* = 0°, 30° and 60°, respectively. For *θ* = 0°, no chiral effect occurs in the metamaterial since there is no cross coupling between two mutually orthogonal electric and magnetic fields. *T*_++_ and *T*_−−_ coincide with each other in Fig. 2(b). Thus, the metamaterial lacks linear polarization conversion and circular dichroism at normal incidence. No polarization conversion dichroism occurs for both the normal and oblique incidence as *T*_+−_ and *T*_−+_ are always identical. Therefore, only *T*_−+_ curves are plotted in Fig. 2(c). However, the oblique incidence leads to extrinsic chirality as a result of the coupling between two orthogonal linearly polarized eigenstates. The strength of the coupling is controlled by the angle of incidence. As the angle of incidence increases to 30°, *T*_*xy*_ reveals a single peak and reaches about 0.13 while the circular dichroism dramatically increases to nearly 0.5 defined as the difference between *T*_++_ and *T*_−−_, meanwhile *T*_−+_ is smaller than that at normal incidence. When the angle of incidence further increases to 60°, it is clearly seen that the linear polarization conversion has an evolution from a single ripple-free peak to a spectrum split into two separated peaks. More importantly, the circular transmittance experiences an intriguing change at *θ* = 60°. The transmittance curves of the LCP and RCP waves are totally separated with high transmittance larger than 0.9 at about 9 GHz and 10 GHz. In addition, the circular polarization conversion is largely suppressed in comparison with the two cases discussed above. At individual resonance, both the transmittance contrasts between the one polarization and the other polarization, the cross-polarization are larger than 10 dB in the simulations. It is noteworthy that the large angle of incidence obviously causes a rapid increase of circular-polarization transmittance and a large suppression of circular-polarization conversion. In addition, the transmittance spectra of the RCP and LCP waves are interchanged when the angle of incidence is reversed to −60°, as a result this extrinsically chiral metamaterial can be used as a circular polarizer.

**Figure 3:**
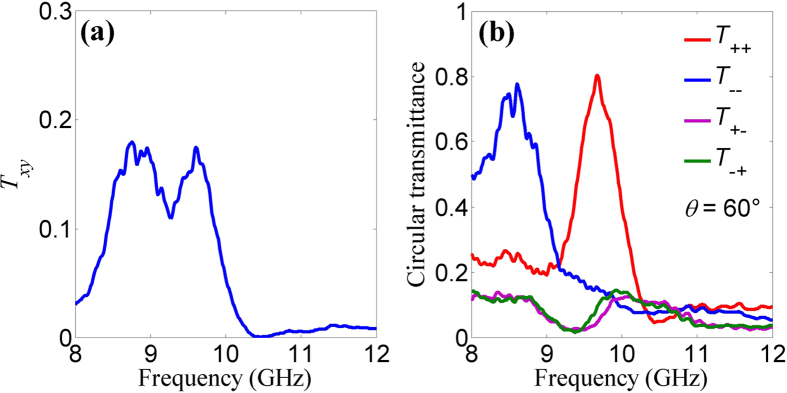
Measured transmittance spectra of the metamaterial at *θ* = 60°. (**a**) Linear polarization conversion and (**b**) circular polarization transmittance.

To verify the performance of the extrinsically chiral metamaterial, we measured the polarization properties of the steel metamaterial sample with a size of 500 × 500 mm^2^, fabricated by laser printing technique. The experiments were carried out in an anechoic chamber using broadband linearly polarized horn antennas (Schwarzbeck BBHA9120D) and a vector network analyzer (Anritsu MS4644A). The experimental data were normalized by incident waves. By changing the orientations of the two horn antennas, all four components of the electromagnetic wave transmission for different polarizations were measured. Further, the circular transmission coefficients can be achieved according to Eq. (1) 11,19,21. The measured transmittance spectra of the linear and circular polarizations at *θ* = 60° are shown in Fig. 3. Obviously, there is a pronounced spectrum split into two peaks for linear polarization conversion *T*_*xy*_, which is consistent with the simulated result in Fig. 2(a). For circular polarization, we observe high transmittance up to 0.8 at both resonances of LCP at about 8.7 GHz and RCP at about 9.7 GHz. In the vicinity of circular resonances, the co-polarization transmittances of *T*_++_ and *T*_−−_ are much larger than the circular polarization conversion of *T*_+−_ and *T*_−+_. At the LCP and RCP resonances, the measured transmittance contrasts between the LCP and RCP waves are about 6 and 7 dB, respectively. The measured results agree with the simulated ones. The difference in amplitude and resonant frequencies may be caused by fabrication errors and random noises in experiments.

**Figure 4:**
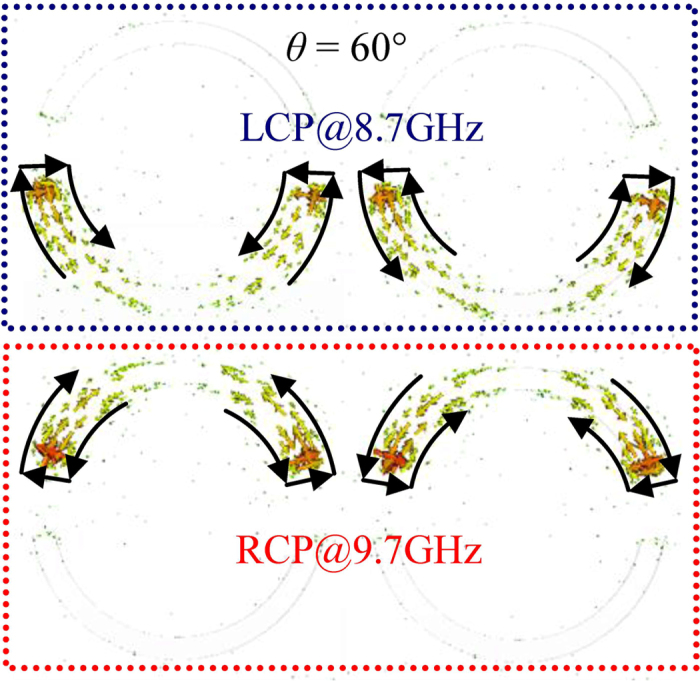
Instantaneous current distributions around two adjacent ASRAs at the resonant modes corresponding to the transmittance peaks in panel (b) of Fig. 2 for *θ* = 60°. The arrows indicate the directions of induced currents.

The amplitude and bandwidth of the linear polarization conversion are affected by both the asymmetry of the ASRA and the angle of incidence^37^. With the increasing angle of incidence, the co-polarization transmittance difference between two orthogonal linearly polarized waves decreases due to weak linear anisotropy while *t*_*xy*_ and *t*_*yx*_ have the same amplitude but opposite phase. Consequently, the circular polarization conversion is largely suppressed, expressed as the formula of *t*_−+_ = *t*_+−_ = (*t*_*xx*_ − *t*_*yy*_)/2. More importantly, the oblique incidence leads to phase lag across the metamaterial surface. For *θ* = 60°, the induced currents of the resonant modes in two adjacent ASRAs are excited in antiphase, while currents have almost the same amplitude shown in Fig. 4. The scattered electromagnetic fields produced by such current configurations are very weak, therefore the transmittances can reach very high values at resonant frequencies for both RCP and LCP incident waves. In addition, short and long apertures are respectively excited by RCP and LCP incident waves. The asymmetry of the ASRA can manipulate the frequency gap between the RCP and LCP resonances, thus we can flexibly design the property of the circular polarizer.

**Figure 5:**
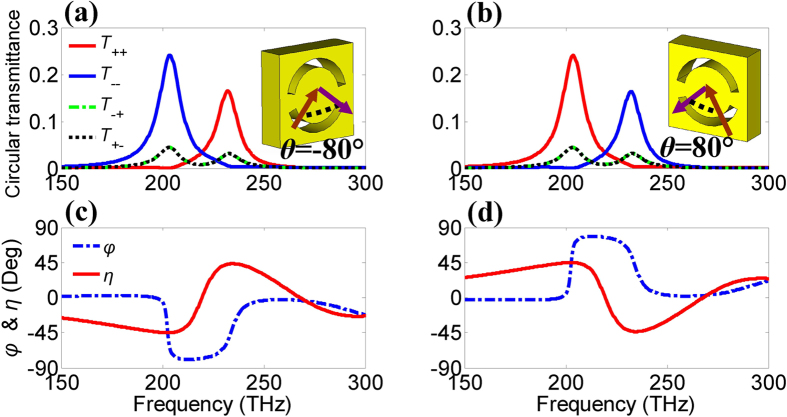
Polarity switching of the optical metamaterial at *θ* = ±80°. (**a,b**) Circular transmittance and (**c,d**) polarization rotation azimuth angle *φ* and ellipticity *η*.

In fact, the underlying mechanism of the dual-polarity circular polarizer can be applied in the optical range. The optical metamaterial has the same pattern as the microwave one shown in the inset of Fig. 5. The ASRA structure is perforated through a freestanding gold film with a thickness of *t* = 200 nm. The other parameters are *d* = 500 nm, *r* = 175 nm, *w* = 50 nm, *α* = 140° and *β* = 165°. The gold nanostructure is modeled using the parameters given in ref. 38. In Fig. 5, the metamaterial exhibits two strong circular resonances at about 205 and 230 THz at *θ* = ±80°, where the transmittance of the one circular polarization is much larger than that of the other one. When the angle of incidence is changed from −80° to 80°, the RCP and LCP resonances can be reversed in Fig. 5(a,b). The polarization rotation azimuth angle *φ* and ellipticity *η* are calculated by Eq. (2) to show polarization state of the transmitted wave, shown in Fig. 5(c,d). At the resonant frequencies of 205 and 230 THz, the ellipticity angles are about 45° and −45°, respectively. The polarization rotation azimuth angle *φ* is up to 80° when *η* = 0°. Evidently, the signs of both the polarization rotation azimuth angle and ellipticity are reversed when the sign of the angle of incidence is reserved. Therefore, the polarity of the circular resonances can be interchangeable by changing the sign of the angle of incidence. Although the angle of incidence is 80°, the circular polarization transmittance is as high as 0.25 and it can be further improved by optimizing the configuration of the optical metamaterial within the scope of easy fabrication.

## Discussion

We have demonstrated a high-efficiency dual-polarity circular polarizer exploiting extrinsic chirality in the single-layer ASRA metamaterial. The strong circular dichroism and optical activity result from the mutual arrangement of the ASRA metamaterial and the incident beam. The large angle of incidence enables the short and long apertures to be individually excited by either of RCP and LCP circular polarizations. At resonant frequencies, the transmitted wave is nearly circularly polarized. The polarity of the circular polarizer can be switched by changing the sign of the angle of incidence. Remarkably, microwave experiments and optical simulations demonstrate that the dual-polarity circular polarizer can work anywhere from microwave to optical frequencies. Such the single-layer metamaterial with extrinsic chirality will be a promising candidate for circular polarization manipulation.

## Methods

Numerical simulations were performed by use of the commercial software CST microwave studio. A lossy metal was simulated with its conductivity of 1 × 10^7^ S/m in the microwave range while the gold nanostructure was modeled by experimental data in the optical range. The transmission properties of the metamaterial were studied for both linearly and circularly polarized waves in the frequency range from 8 to 12 GHz. The steel metamaterial sample with a size of 500 × 500 mm^2^ was fabricated by laser printing technique. The experiments were carried out in an anechoic chamber using broadband linearly polarized horn antennas (Schwarzbeck BBHA9120D) and a vector network analyzer (Anritsu MS4644A). The experimental data were normalized by incident waves. By changing the orientations of the two horn antennas, all four components of the electromagnetic wave transmission for different polarizations were measured. Further, the circular transmission coefficients can be achieved according to Eq. (1) 11,19,21.

## Additional Information

**How to cite this article**: Shi, J. H. *et al.* Dual-polarity metamaterial circular polarizer based on giant extrinsic chirality. *Sci. Rep.* **5**, 16666; doi: 10.1038/srep16666 (2015).
